# Supplementary material for: Protein quaternary structures in solution are a mixture of multiple forms
Source: Chem Sci. 2022 Sep 21;13(39):11680–95. doi: 10.1039/d2sc02794a (PMC9555727; doi:10.1039/d2sc02794a)
Supplement: SC-013-D2SC02794A-s001 [file SC-013-D2SC02794A-s001.pdf]

Electronic Supplementary Material (ESI) for Chemical Science.
This journal is © The Royal Society of Chemistry 2022

Supplemental information

Protein Quaternary Structures in Solution are a Mixture of Multiple forms

Shir Marciano, Debabrata Dey, Dina Listov, Sarel J. Fleishman, Adar Sonn-Segev, Haydyn Mertens, Florian Busch, Yongseok Kim, Sophie R. Harvey, Vicki H. Wysocki, Gideon Schreiber.

Supplemental Figures 1-8
Supplemental Tables 1-3

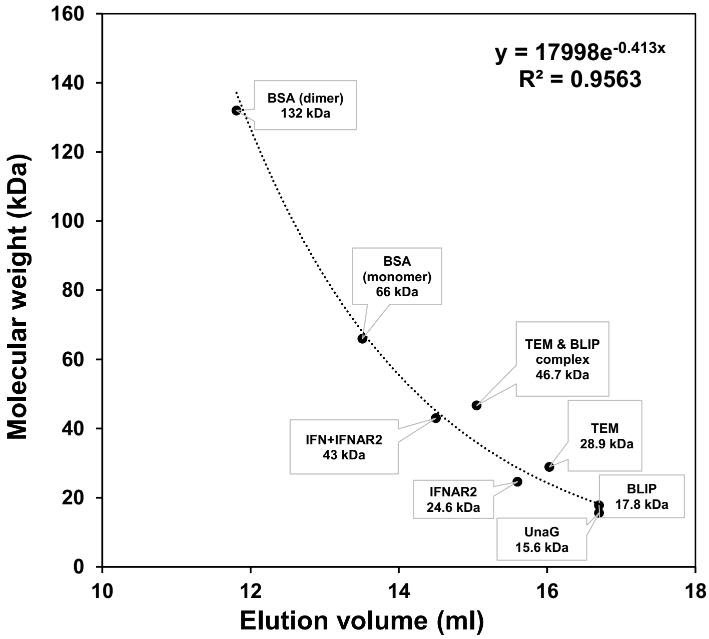

Figure S1- Standards calibration curve fit for known proteins elution volumes and molecular weights. The proteins (from largest to smallest - elution volume (ml), MM (kDa)). BSA dimer (11.8, 132.8), BSA monomer (13.5, 66.4), IFN+IFNAR2 (14.5, 44.0), TEM & BLIP (15.1, 46.7), IFNAR2 (15.6, 24.7), TEM (16.0, 28.9), BLIP (16.7, 17.8), UnaG (16.7, 15.6). The data fitted best an exponential, which was used to calculate the MW of unknown proteins.

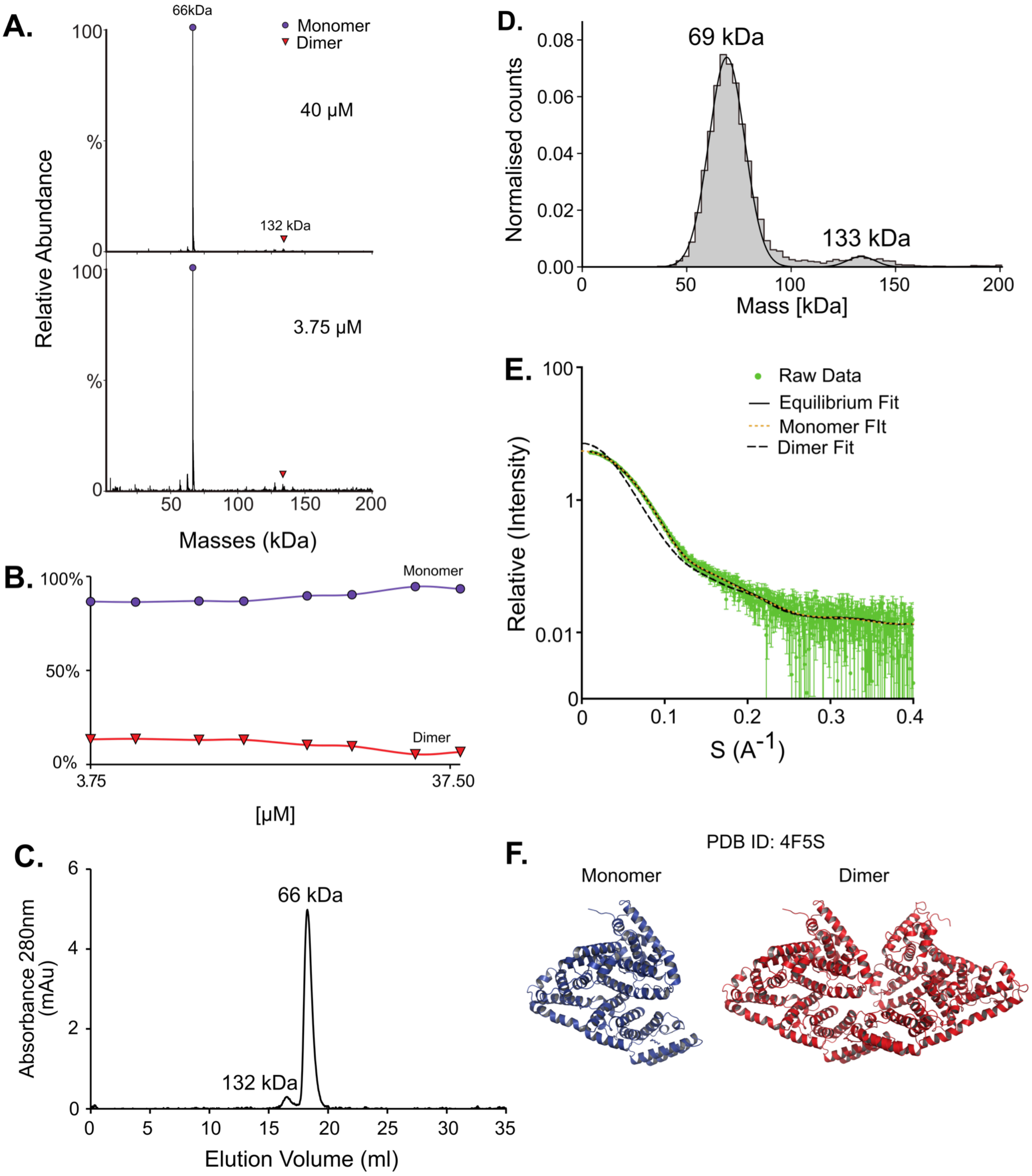

Figure S2- BSA is mostly a monomer with some small dimeric fraction. Measurements of BSA, a well known protein, in all the different methods results in similar quaternary structure- mostly a monomer with small dimeric fraction. **A.** Native MS results shows one main peak that corresponds to a 66 kDa monomer and a small peak of 132 kDa dimer in a ratio that is not concentration dependent. **B.** Native MS in a range of protein concentrations, 3.75 µM - 40 µM, shows the proteins oligomeric state to be independent on the concentration (see also fig. S3). **C**. SEC analysis shows two peaks- the small one eluted at 11.8 ml corresponds to 132 kDa (a dimer) and the second, main one, eluted at 13.5 ml corresponds to the monomeric form of BSA at 66 kDa. **D**. Mass photometry measurements of the protein show masses that fit a monomer and a dimer- 69 kDa and 133 kDa. **E.** SAXS measurements were done in one concentration of 27 µM and shows that more than 90% of the protein is in monomeric form. SAXS equilibrium fitting using the program OLIGOMER and PDB id: 4F5S shows that the data is well fitted with the equilibrium and monomer but poorly with the dimeric fit (black dashed line). **F.** Assemblies of BSA using OLIGOMER and the fit.

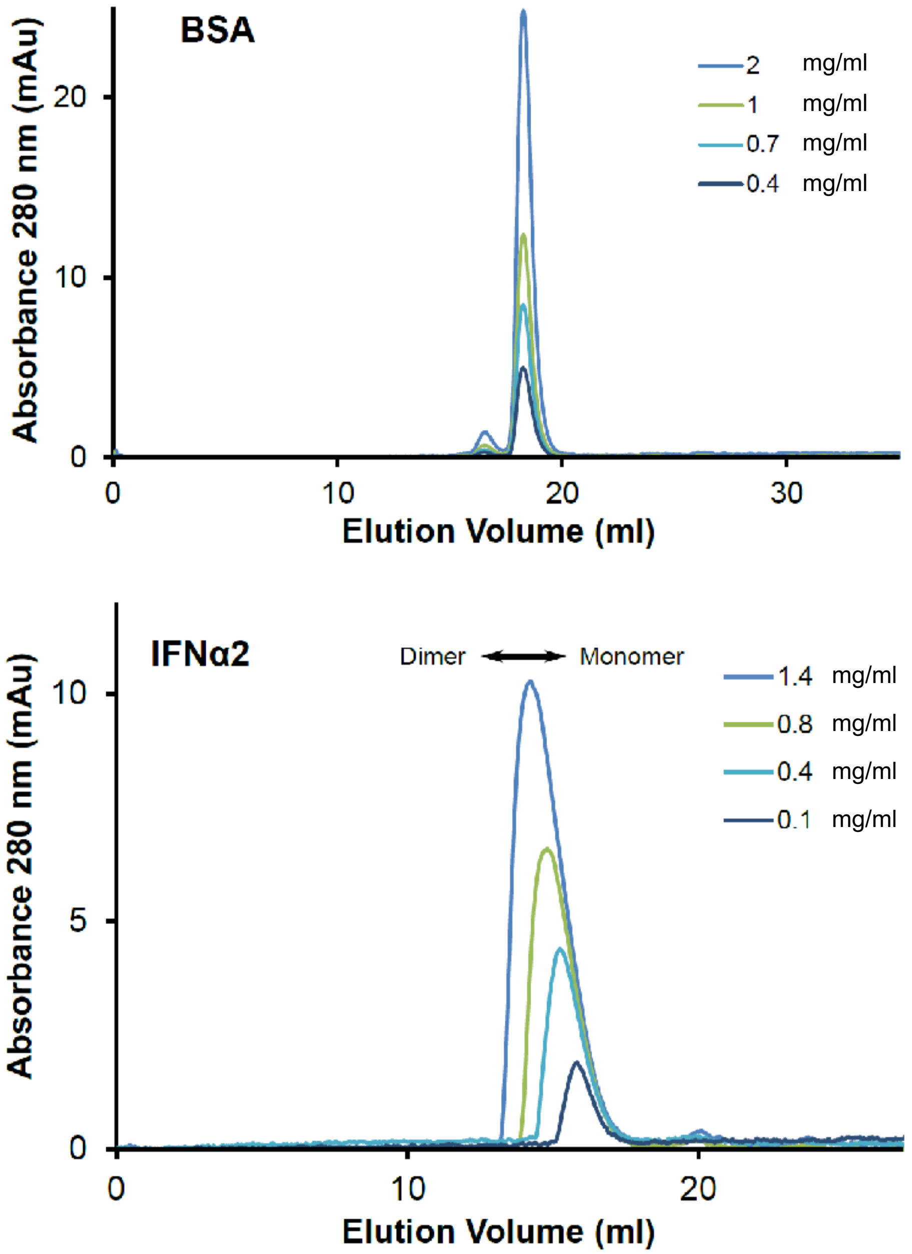

Figure S3- SEC concentration-dependent elution of BSA and IFNα2. SEC analysis of BSA at 0.4-2 mg/ml and IFNα2 0.1-1.4 mg/ml shows that BSA's elutes at the same volume, whereas IFNα2 elution volume decreases with increasing concentration. This suggests a concentration dependent oligomerization of IFNα2.

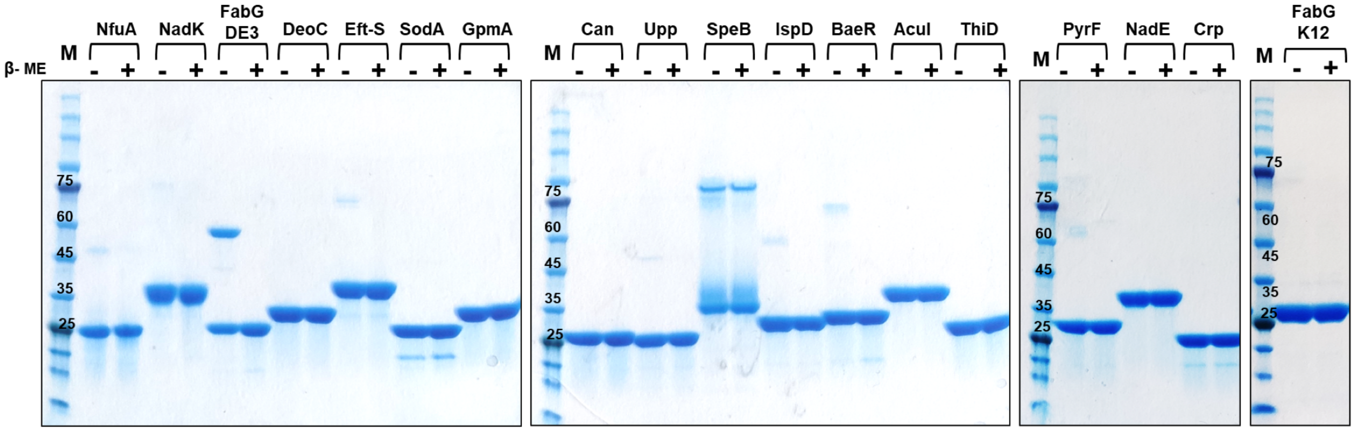

Protein's name	# of cys residues	Protein's name	# of cys residues	Protein's name	# of cys residues	Protein's name	# of cys residues
NfuA	4	Eft-s	2	SpeB	4	PyrF	3
NadK	6	SodA	0	IspD	5	NadE	3
FabG^{DE3}	1	GpmA	0	BaeR	4	CRP	3
FabG^{K12}	0	Can	5	AcuI	3		
DeoC	4	Upp	1	ThiD	3		

Figure S4- SDS-PAGE analysis of all proteins with and without reducing agent- β-mercaptoethanol. The gel represents each protein with and without the addition of β-mercaptoethanol prior to loading to the gel. The table represents the number of cysteine residues in each protein. The gel shows that the only protein where inter-disulfide bridges were formed is FabG^{DE3}, where almost 50% of the protein is in inter-protein disulfide bonded state, while for the other proteins the dominant form is the same with and without reducing agent. FabG^{K12} does not show this as the protein does not contain any cys residue.

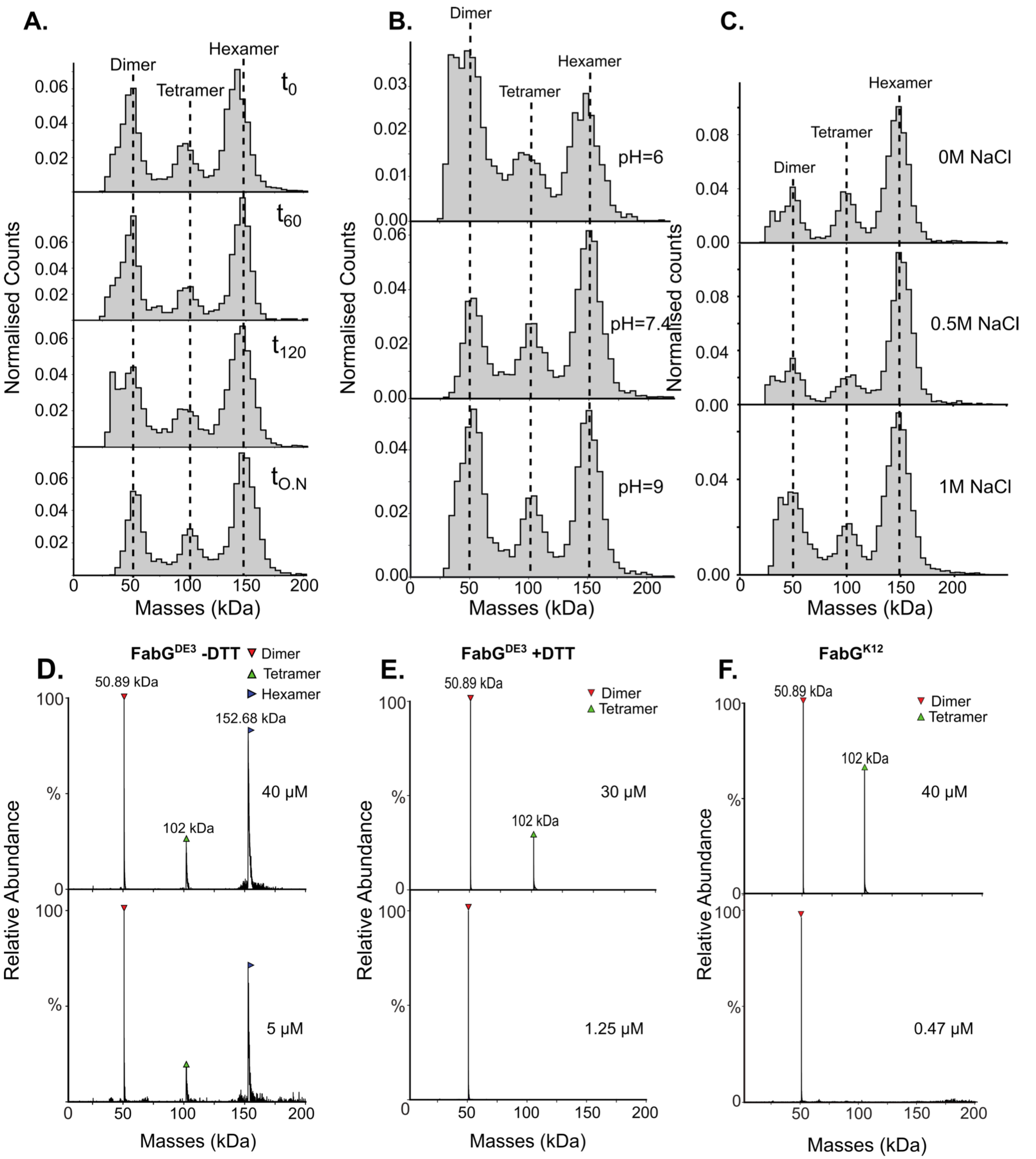

Figure S5- FabG^{DE3} oligomerizations state equilibrium is not affected by protein-dilution or buffer. FabG^{DE3} oligomerization state was determined at a concentration of 38 nM by MP. **A**. Measurements of time points after dilution from 60 μM of FabG^{DE3} shows similar oligomeric states at all time points. **B.** FabG^{DE3} oligomerization states at pH=6 (50 mM Sodium Citrate, 50 mM NaCl pH=6), pH=7.4 (PBS) and pH=9 (50 mM Tricine , 50 mM NaCl pH=9). Overall, the changes in the fraction of the different oligomeric states between pH 6-9 are small. **C**. Salt dependence of the oligomerization state of FabG^{DE3} : 0 M, 500 mM and 1M NaCl in 50 mM HEPES buffer, pH 7.4 were used. FabG has shown a similar ration between hexameric, tetrameric and dimeric forms at all three salt concentrations. **D.** and **E.** are nMS measurements of FabG^{DE3} (without (D) or with (E) DTT. **F**. nMs of FabG^{K12} in high and low protein concentration.

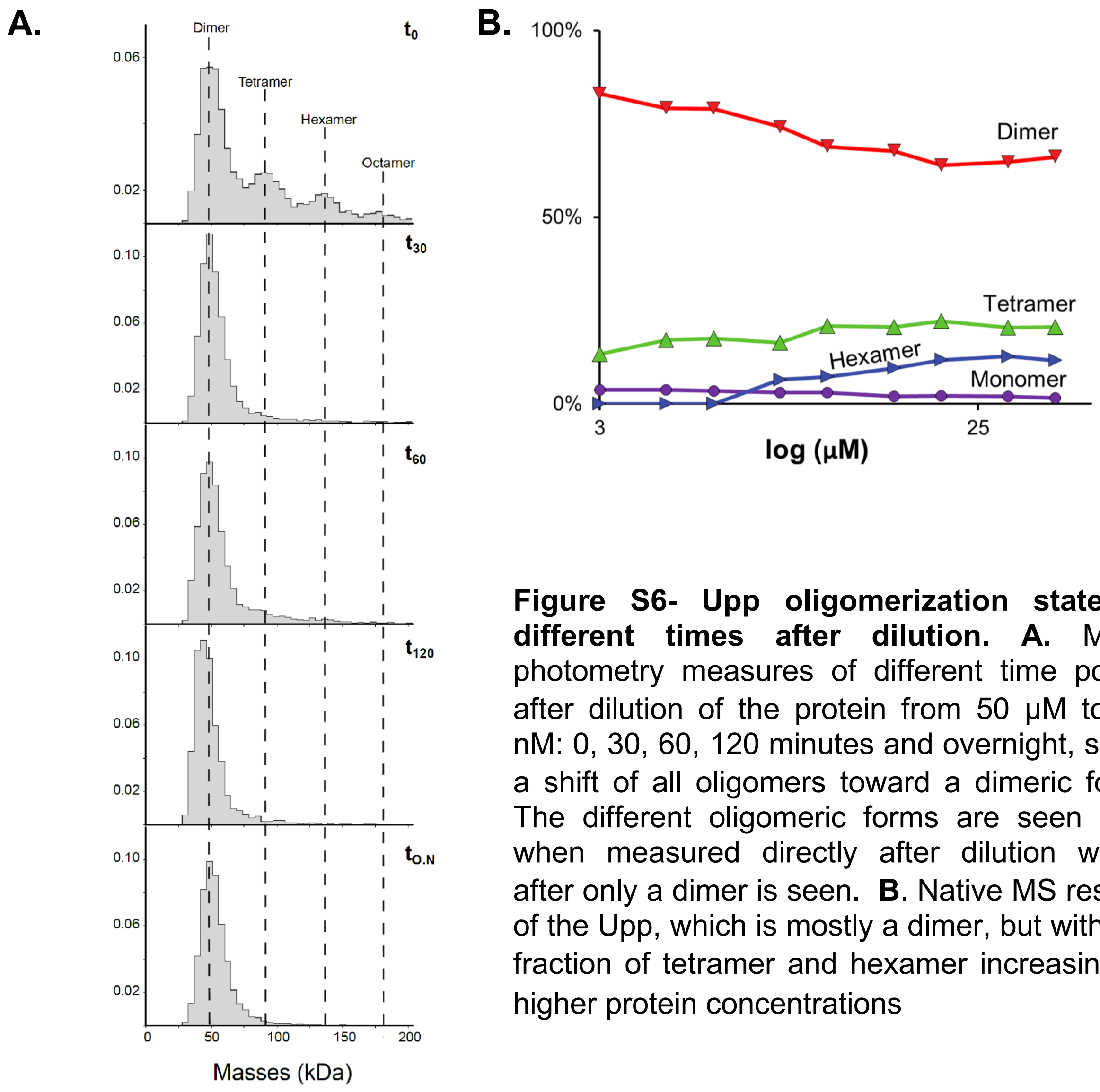

Figure S6- Upp oligomerization state at different times after dilution. A. Mass photometry measures of different time points after dilution of the protein from 50 µM to 50 nM: 0, 30, 60, 120 minutes and overnight, show a shift of all oligomers toward a dimeric form. The different oligomeric forms are seen only when measured directly after dilution which after only a dimer is seen. **B**. Native MS results of the Upp, which is mostly a dimer, but with the fraction of tetramer and hexamer increasing at higher protein concentrations

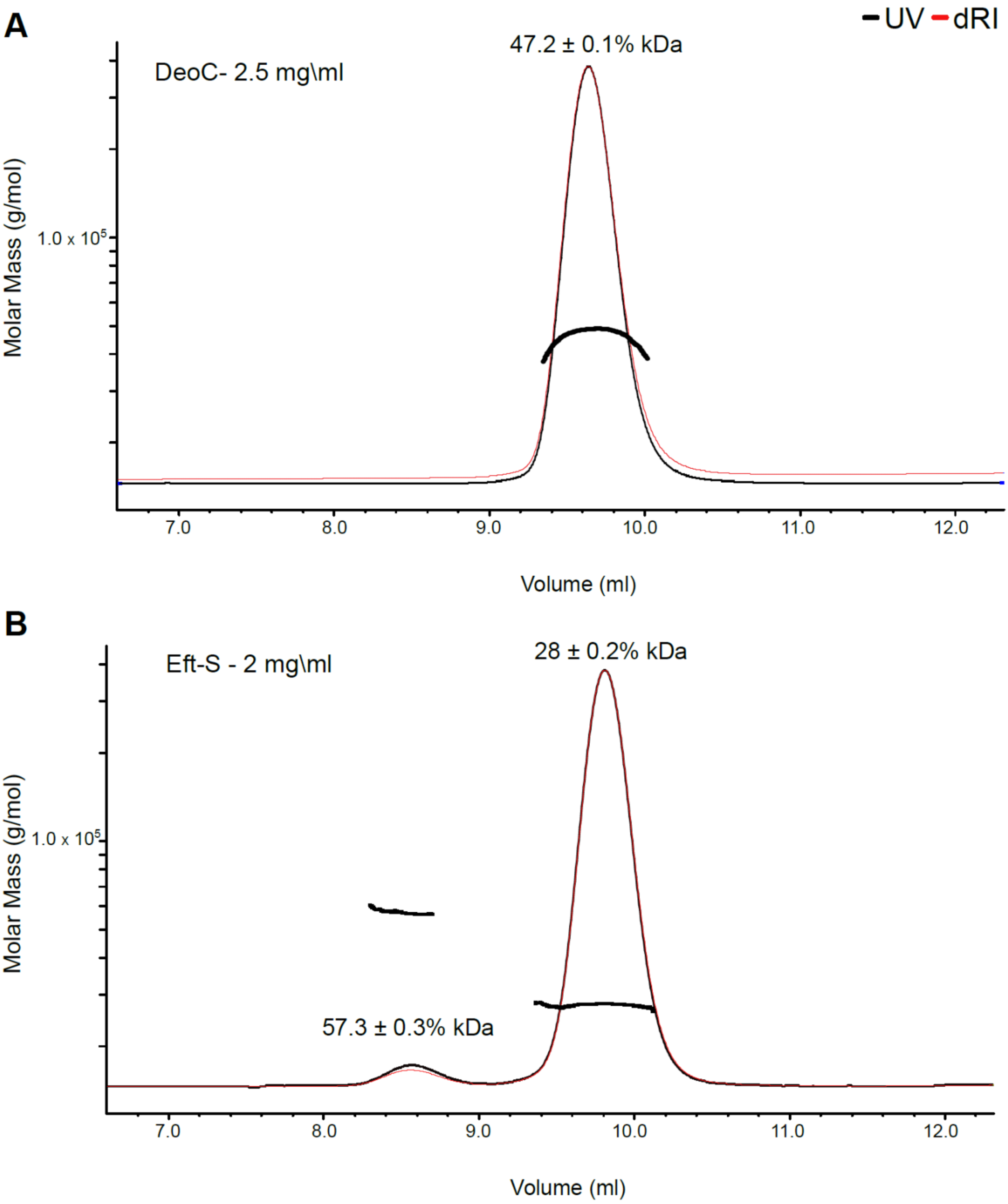

Figure S7- SEC-MALS of E-fts and DeoC. A. DeoC is eluted as a single peak, with MALS-detector measureing a MM of 47.2 kDa. As this MM does not corresponds to a monomer (27.7 kDa) or a dimer (55 kDa), we conclude that the peak is a mixture of both. **B.** Eft-S is eluted in two peaks, a minor dimeric peak corresponding to 57.3 kDa and a major monomeric peak corresponding to 28 kDa.

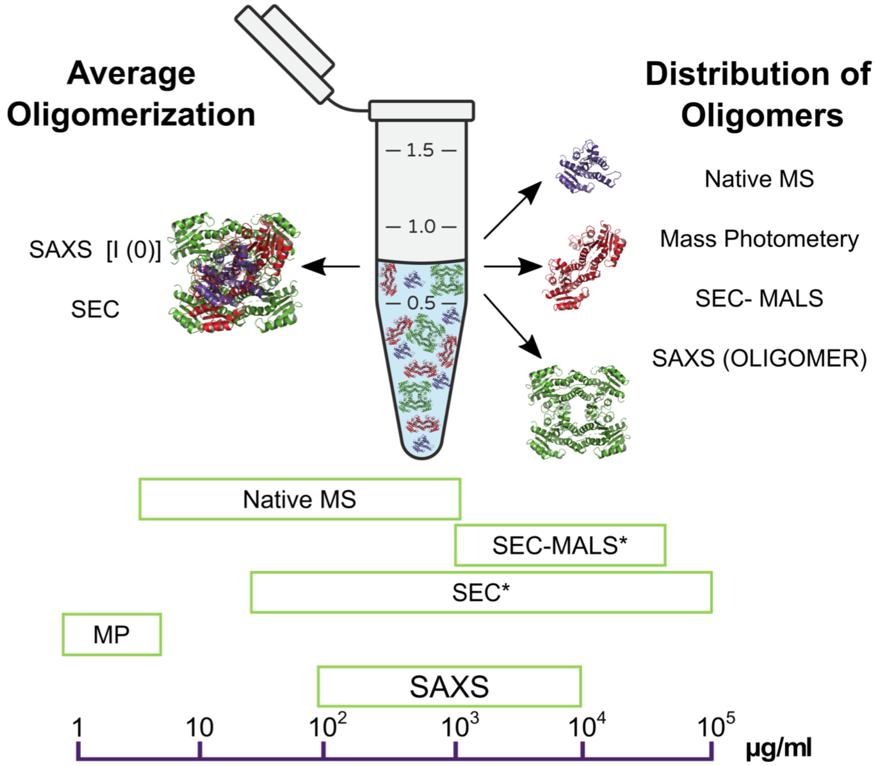

Figure S8- Graphical summary representation of the different methods to determine oligomerization. Comparing the different methods for determining oligomerization composition of a protein. Each method is suitable for different protein concentrations. *I(0)* from SAXS as well as SEC give information of the average oligomerization state, whereas, native MS, MP, SEC-MALS (depending on the equilibrium of the different oligomers), and SAXS (by using OLIGOMER) determine the distribution of the oligomers in solution. The (*) in the SEC methods represent the injected concentration that is diluted during the run of the SEC. The ruler of µg/ml represents protein concentrations applicable for the different methods.

TABLE S1

Summary table of small-angle X-ray scattering results

Sample	*Conc. (mg/ml)*	R_g (Å)	d_{max} (Å)	M_r from $I(0)$ (Da) (ratio to predicted value)
SodA	0.25	21.2 ± 0.2	70 ± 5	52542 (2.3)
	0.51	22.7 ± 0.1	75 ± 5	48500 (2.1)
	1.01	22.7 ± 0.1	75 ± 5	45806 (2.0)
	2.03	22.7 ± 0.1	72 ± 5	45806 (2.0)
DeoC	0.26	25.2 ± 0.3	85 ± 5	55606 (2.0)
	0.52	25.7 ± 0.1	85 ± 5	54216 (2.0)
	1.04	26.1 ± 0.1	85 ± 5	51436 (1.9)
	2.08	26.0 ± 0.1	80 ± 5	50046 (1.8)
$FabG^{DE3}$	0.24	33.2 ± 0.1	100 ± 5	89174(3.5)
	0.48	33.7 ± 0.1	100 ± 5	91961 (3.6)
	0.95	33.9 ± 0.1	105 ± 5	91961 (3.6)
	1.90	34.0 ± 0.2	106 ± 5	93354 (3.7)
NadK	0.25	36.2 ± 0.3	11.5 ± 5	77202 (2.4)
	0.5	3.7 ± 0.2	12 ± 5	81413 (2.5)
	1.0	38.4 ± 0.1	12 ± 5	87028 (2.7)
	2.0	39.8 ± 0.1	12.5 ± 5	87828 (2.7)

TABLE SAXS1A

Small-angle X-ray scattering parameters and results SodA, DeoC, $FabG^{DE3}$

(*a*) Sample details			
	SodA	DeoC	$FabG^{DE3}$
Organism	*Escherichia coli (strain K12)*	*Escherichia coli (strain K12)*	*Escherichia coli (strain K12)*
Source	*Escherichia coli BL21 (DE3)*	*Escherichia coli BL21 (DE3)*	*Escherichia coli BL21 (DE3)*
UniProt sequence ID (residues in construct)	P00448	P0A6L0	P0AEK2
Extinction coefficient ε (280 nm, 0.1% w/v)	1.893	0.523	0.452

Partial specific volume $\bar{\upsilon}$ ($cm^3 g^{-1}$)	0.737	0.741	0.742
Mean solute and solvent scattering length densities and mean scattering contrast $\Delta\bar{\rho}$ ($\rho_{protein}$-$\rho_{solvent}$) (10^{10} cm^{-2})	2.87 (12.297-9.429)	2.81 (12.238-9.429)	2.80 (12.231-9.429)
Molecular mass *M* from chemical composition (monomer) (Da)	22950	27619	25377
Sample concentration (mg ml^{-1}) [A280nm]	0.25-2.0	0.26-2.08	0.24-1.90
Sample volume (ul)		40	
Solvent composition		50 mM HEPES pH 7.2	

(*b*) SAS data collection parameters

Instrument/Data processing	EMBL P12 (PETRA-III, DESY, Hamburg) with Pilatus6M detector (Blanchet et al. 2015)
Wavelength (Å)	1.24
Beam geometry (size, sample-to-detector distance)	0.12 × 0.25 mm^2, 3.0 m
s-measurement range ($Å^{-1}$)	0.002-0.5
Absolute scaling method	Comparison with scattering from 1.2 mm pure H_2O
Basis for normalization to constant counts	To transmitted intensity by beam-stop counter
Method for monitoring radiation damage	Frame comparison
Exposure time, number of exposures	1.8 s (40 × 0.045 s)
Sample temperature (°C)	20

(*c*) Software employed for SAS data reduction, analysis and interpretation

SAS data reduction	*I(s)* versus *s* using *RADAVER* (ATSAS 2.8.3; Petoukhov et al., 2012), solvent subtraction using *PRIMUSqt* (ATSAS 2.8.3; Petoukhov et al., 2012)
Calculation of ε from sequence	*ProtParam* (Gasteiger et al., 2005)
Calculation of $\Delta\bar{\rho}$ and $\bar{\upsilon}$ values from chemical composition	Direct Calculation (in-house routines) (Fraser et al. 1978)
Basic analyses: Guinier, *P*(*r*), scattering particle volume (V_P)	*PRIMUSqt* from ATSAS 2.8.3 (Petoukhov *et al.*, 2012)
Equilibrium analysis	OLIGOMER (Konarev et al., 2003)
Atomic structure modelling	CRYSOL (Svergun et al., 1995), SASREF (Petoukhov et al., 2005)
Molecular graphics	PyMOL v2.3 MacOS 10.13.6

(*d*) Structural parameters[a]

Guinier Analysis	SodA	DeoC	$FabG^{DE3}$
I(0) (cm^{-1})	0.034 ± 0.001	0.036 ± 0.001	0.067 ± 0.001
R_g (Å)	22.8 ± 0.1	25.6 ± 0.1	35.5 ± 0.1
q-range ($Å^{-1}$)	0.012-0.057	0.011-0.051	0.016-0.036
M_r from *I*(0) (Da) (ratio to predicted value)	45806 (2.0)	50046 (1.8)	93354 (3.7)
***P*(*r*) analysis**	**SodA**	**DeoC**	**$FabG^{DE3}$**
I(0) (cm^{-1})	0.034 ± 0.001	0.036 ± 0.001	0.066 ± 0.001
R_g (Å)	22.7 ± 0.1	26.0 ± 0.1	34.0 ± 0.1
d_{max} (Å)	72.2 ± 5	80.0 ± 5	106 ± 5
q-range ($Å^{-1}$)	0.012-0.287	0.011-0.287	0.016-0.287
χ^2 (total estimate from *GNOM*)	1.0 (0.94)	1.1 (0.93)	1.1 (0.83)
M_r from *I*(0) (Da) (ratio to predicted value)	45967 (2.0)	50477 (1.8)	91264 (3.6)
Volume(V_P) ($Å^3$)	53629	61429	212014
M_r from V_P (Da) (ratio to predicted value)	33518 (1.5)	38393 (1.4)	132509 (5.2)

(*e*) Equilibrium modeling results

OLIGOMER fitting	SodA	DeoC	$FabG^{DE3}$

Starting crystal structures	-	1KTN	1I01
Multimers used	-	Dimer, monomer	Hexamer, tetramer, dimer, monomer
q-range for fitting (Å)	-	0.014-0.359	0.014-0.359
χ^2, *CORMAP P* value	-	1.2-1.7 (0.000)	1.2-1.5 (0.000-0.260)
(*e*) Single model calculation results			
CRYSOL fitting	SodA	DeoC	$FabG^{DE3}$
Crystal structure	1D5N	-	-
q-range for fitting (Å)	0.014-0.359	-	-
χ^2, *CORMAP P* value	1.0-1.2 (0.009-0.037)	-	-
(f) SASBDB IDs for data and models			
	SodA	DeoC	$FabG^{DE3}$
	SASDLP4	SASDLQ4	SASDLR4

[a]parameters reported for highest sample concentration

TABLE SAXS1B

Small-angle X-ray scattering parameters and results for NadK

(*a*) Sample details	
	NadK
Organism	*Escherichia coli (strain K12)*
Source	*Escherichia coli BL21 (DE3)*
UniProt sequence ID (residues in construct)	P0A7B3
Extinction coefficient ε (280 nm, 0.1% w/v)	0.750
Partial specific volume $\bar{\upsilon}$ ($cm^3\ g^{-1}$)	0.742
Mean solute and solvent scattering	2.79 (12.220-9.429)

length densities and mean scattering contrast $\Delta\bar{\rho}$ ($\rho_{protein}$-$\rho_{solvent}$) (10^{10} cm^{-2})	
Molecular mass *M* from chemical composition (monomer) (Da)	32566
Sample concentration (mg ml^{-1}) [A280nm]	0.25-2.0
Sample volume (ul)	40
Solvent composition	50 mM HEPES pH 7.2
(*b*) SAS data collection parameters	
Instrument/Data processing	EMBL P12 (PETRA-III, DESY, Hamburg) with Pilatus6M detector (Blanchet et al. 2015)
Wavelength (Å)	1.24
Beam geometry (size, sample-to-detector distance)	0.12 × 0.25 mm^2, 3.0 m
s-measurement range ($Å^{-1}$)	0.002-0.5
Absolute scaling method	Comparison with scattering from 1.2 mm pure H_2O
Basis for normalization to constant counts	To transmitted intensity by beam-stop counter
Method for monitoring radiation damage	Frame comparison
Exposure time, number of exposures	1.8 s (40 × 0.045 s)
Sample temperature (ºC)	20
(*c*) Software employed for SAS data reduction, analysis and interpretation	
SAS data reduction	*I(s)* versus *s* using *RADAVER* (ATSAS 2.8.3; Petoukhov et al., 2012), solvent subtraction using *PRIMUSqt* (ATSAS 2.8.3; Petoukhov et al., 2012)
Calculation of ε from sequence	*ProtParam* (Gasteiger et al., 2005)

Calculation of $\Delta\bar{\rho}$ and $\bar{\upsilon}$ values from chemical composition	Direct Calculation (in-house routines) (Fraser et al. 1978)
Basic analyses: Guinier, *P*(*r*), scattering particle volume (V_P)	*PRIMUSqt* from ATSAS 2.8.3 (Petoukhov *et al*., 2012)
Equilibrium analysis	OLIGOMER (Konarev et al., 2003)
Atomic structure modelling	CRYSOL (Svergun et al., 1995), SASREF (Petoukhov et al., 2005)
Molecular graphics	PyMOL v2.3 MacOS 10.13.6

(*d*) Structural parameters[a]

Guinier Analysis	NadK
I(0) (cm^{-1})	0.0630 ± 0.001
R_g (Å)	40.3 ± 0.2
q-range ($Å^{-1}$)	0.1474-0.3143
M_r from *I*(0) (Da) (ratio to predicted value)	88432 (2.7)
P(*r*) analysis	NadK
I(0) (cm^{-1})	0.0626 ± 0.001
R_g (Å)	39.8 ± 0.1
d_{max} (Å)	12.5 ± 5
q-range ($Å^{-1}$)	0.1474-2.8732
χ^2 (total estimate from *GNOM*)	1.2 (0.89)
M_r from *I*(0) (Da) (ratio to predicted value)	87828 (2.7)
Volume(V_P) ($Å^3$)	259247
M_r from V_P (Da) (ratio to predicted value)	162029 (5.0)

(*e*) Equilibrium modeling results

OLIGOMER fitting	NadK
Starting crystal structures	4HAO
Multimers used	8-mer, tetramer, dimer, monomer
q-range for fitting (Å)	0.014-0.359

χ^2, *CORMAP P* value	1.2-1.5 (0.00-0.01)
(f) SASBDB IDs for data and models	
	NadK
	SASDMT3

[a]parameters reported for highest sample concentration

References

Bienert, S., Waterhouse, A., de Beer, T.A., Tauriello, G., Studer, G., Bordoli, L. and Schwede, T., 2017. The SWISS-MODEL Repository—new features and functionality. *Nucleic acids research*, *45*(D1), pp.D313-D319.

Blanchet, C.E., Spilotros, A., Schwemmer, F., Graewert, M.A., Kikhney, A., Jeffries, C.M., Franke, D., Mark, D., Zengerle, R., Cipriani, F., et al. (2015). Versatile sample environments and automation for biological solution X-ray scattering experiments at the P12 beamline (PETRA III, DESY). J. Appl. Crystallogr. *48*, 431–443.

Franke, D., and Svergun, D.I. (2009). DAMMIF, a program for rapid ab-initio shape determination in small-angle scattering. J. Appl. Crystallogr. *42*, 342–346.

Franke, D., Kikhney, A.G., and Svergun, D.I. (2012). Automated acquisition and analysis of small angle X-ray scattering data. Nucl. Instrum. Methods Phys. Res. Sect. Accel. Spectrometers Detect. Assoc. Equip. *689*, 52–59.

Franke, D., Petoukhov, M.V., Konarev, P.V., Panjkovich, A., Tuukkanen, A., Mertens, H.D.T., Kikhney, A.G., Hajizadeh, N.R., Franklin, J.M., Jeffries, C.M., et al. (2017). ATSAS 2.8: a comprehensive data analysis suite for small-angle scattering from macromolecular solutions. J. Appl. Crystallogr. *50*, 1212–1225.

Fraser, R. D. B.,MacRae, T. P. & Suzuki, E. (1978). J. Appl. Crystallogr. 11, 693-694

Guinier, A. SMALL-ANGLE SCATTERING OF X-RAYS. 276.

Konarev, P.V., Volkov, V.V., Sokolova, A.V., Koch, M.H. and Svergun, D.I., 2003. PRIMUS: a Windows PC-based system for small-angle scattering data analysis. *Journal of applied crystallography*, *36*(5), pp.1277-1282.

E. Krissinel and K. Henrick (2007). 'Inference of macromolecular assemblies from crystalline state.'. J. Mol. Biol. 372, 774--797.

Panjkovich, A., and Svergun, D.I. (2018). CHROMIXS: automatic and interactive analysis of chromatography-coupled small-angle X-ray scattering data. Bioinforma. Oxf. Engl. *34*, 1944–1946.

Petoukhov, M.V. and Svergun, D.I. (2005) Global rigid body modelling of macromolecular complexes against small-angle scattering data. *Biophys. J.*, **89**, 1237-1250

Petoukhov, M.V., Franke, D., Shkumatov, A.V., Tria, G., Kikhney, A.G., Gajda, M., Gorba, C., Mertens, H.D.T., Konarev, P.V., and Svergun, D.I. (2012). New developments in the ATSAS program package for small-angle scattering data analysis. J. Appl. Crystallogr. *45*, 342–350.

Semenyuk, A.V., and Svergun, D.I. (1991). GNOM – a program package for small-angle scattering data processing. J. Appl. Crystallogr. *24*, 537–540.

Svergun, D., Barberato, C., and Koch, M.H.J. (1995). *CRYSOL* – a Program to Evaluate X-ray Solution Scattering of Biological Macromolecules from Atomic Coordinates. J. Appl. Crystallogr. *28*, 768–773.

Table S2 - Results summary table of 17 *E.Coli* proteins

NfuA- P63020

Method	MS conc.	MS, kDa	MP, kDa (%)	SAXS, kDa	SEC, kDa	Swiss model calc.	Uniprot (kDa)	PDB	Protein abundance	
Concentrations	0.4 - 40 µM		21 nM	9-72 µM 0.2-1.5mg\ml	14.33 µM				Ref.1	Ref.2
Monomer		20.937	below treshold	$I(0)$ = 39.2 (σ=3.5)	31 (24-40)	Monomer	20.930		10200	3850
Dimer			37 (95%)				Homodimer			
Graph	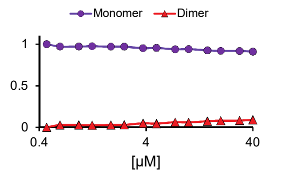		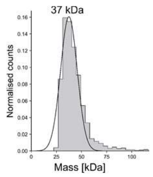		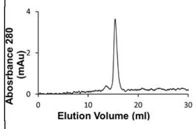			NA	highly expressed protein	

NadK- P0A7B3

Method	MS conc.	MS, kDa	MP, kDa (%)	SAXS, kDa	SEC, kDa	Swiss model calc.	Uniprot (kDa) and oligomerization	PDB	Protein abundance (copy number	
Concentrations	1.25 - 40 µM		88 nM	8-61 µM 0.25-2mg\ml	9.2 µM				Ref.1	Ref.2
Monomer		32.51					32.57			
Dimer		65.01	63 (46%)	$I(0)$ =83.5 (σ=5.2)		Dimer				
Tetramer		130.02	123 (39%)		88 (69-113)					
Hexamer			190 (2%)				Homohexamer			
Octamer			248 (2%)							
Graph	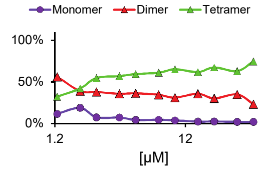		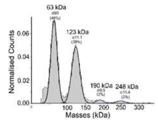	OLIGOMER software fit: 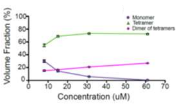	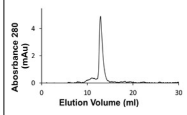			4HAO (similar in 82.5%)	NA	

FabG^{DE3}- P0AEK2

Method	MS conc.	MS, kDa	MP, kDa (%)	SAXS, kDa	SEC, kDa	Swiss model calc.	Uniprot (kDa) and oligomerization	PDB	Protein abundance (copy number	
concentrations	1.87 - 40 µM		38 nM	9-74 µM 0.24-1.9 mg\ml	11.74 µM				Ref.1	Ref.2
Monomer							25.56		13800	6053
Dimer		50.89	51 (32%)							
Tetramer		102	100 (22%)	$I(0)$ =91.6 (σ=1.8)	101 (79-129)	Homotetramer	Homotetramer			
Hexamer		152.68	148 (38%)							
Graph	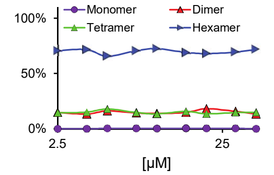		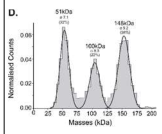	OLIGOMER software fit: 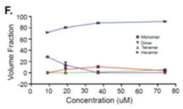	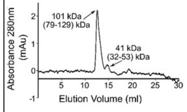			1I01 - homotetramer 1q7b- homotetramer 1q7c- homotetramer	highly expressed protein	

DeoC - P0A6L0

Method	MS conc.	MS, kDa	MP, kDa (%)	SAXS, kDa	SEC, kDa	Swiss model calc.	Uniprot (kDa) and oligomerization	PDB	Protein abundance (copy number	
concentrations	0.17 - 40 µM		53 nM	9-75 µM 0.3-2 mg\ml	10.8 µM				Ref.1	Ref.2
Monomer		27.69	36 (68%) fitted: 35(52%)		45 (35-57)	Monomer and homodimer	27.74 Monomer and		67100	6908
Dimer		55.38	52 (58%) fitted: 52(48%)	$I(0)$ =52.8 (σ=2.5)						
Graph	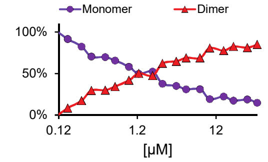		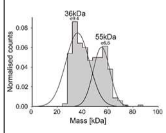	OLIGOMER software fit: 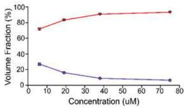	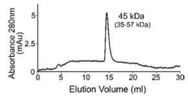			1KTN 1JCJ 5EMU	highly expressed protein	

Eft-S - P0A6P1

Method	MS conc.	MS, kDa	MP, kDa (%)	SAXS, kDa	SEC, kDa	Swiss model calc.	Uniprot (kDa) and oligomerization	PDB	Protein abundance (copy number	
concentrations	0.94 - 40 µM			7-56uM<br>0.2-1.7 mg\ml	9.9 µM				Ref.1	Ref.2
Monomer		30.36		$I(0)$ = 64.7 (σ=5.2)	41 (32-53)	monomer	30.29		66000	14933
Dimer						Dimer- hetromer	Dimer			
Tetramer										
Graph	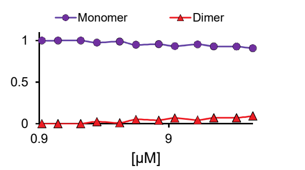		NA<br>Under the detection limit of MP		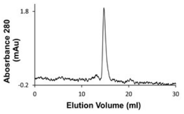			1EFU	highly expressed protein	

SodA- P00448

Method	MS conc.	MS, kDa	MP, kDa (%)	SAXS, kDa	SEC, kDa	Swiss model calc.	Uniprot (kDa) and oligomerization	PDB	Protein abundance (copy number	
concentrations	0.94 - 40 µM		150 nM	11-88 µM<br>0.25-2 mg\ml	1.36 µM				Ref.1	Ref.2
Monomer					36 (28-45)		22.97		36900	11930
Dimer		46.04	52 (97%)	$I(0)$ = 48.2 (σ=3.2)		Dimer	Homodimer			
Graph	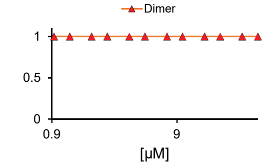		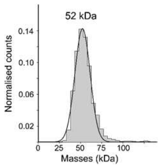	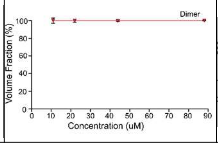	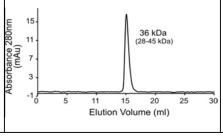			1IXB 1D5N<br>1VEW	highly expressed protein	

GpmA- P62707

Method	MS conc.	MS, kDa	MP, kDa (%)	SAXS, kDa	SEC, kDa	Swiss model calc.	Uniprot (kDa) and oligomerization	PDB	Protein abundance (copy number	
concentrations	0.156 - 40 µM		128 nM	10-77 µM<br>0.3-2 mg\ml	10.55 µM				Ref.1	Ref.2
Monomer							28.43		14400	6169
Dimer		56.99	59 (80%)	$I(0)$ = 68.7 (σ=4.7)		Homodimer	Homodimer			
Tetramer		114.00	116 (4%)							
Graph	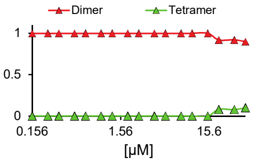		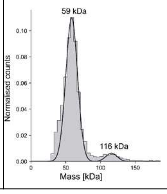		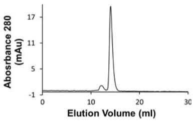			1E58	highly expressed protein	

Can- P61517

Method	MS conc.	MS, kDa	MP, kDa (%)	SAXS, kDa	SEC, kDa	Swiss model calc.	Uniprot (kDa) and oligomerization	PDB	Protein abundance (copy number	
concentrations	0.938 - 40 µM		60 nM	10-83 µM<br>0.3-2 mg\ml	12 µM				Ref.1	Ref.2
Monomer		25.10					25.1		4940	1611
Dimer				$I(0)$ = 86.4 (σ=5.3)			Homodimer			
Trimer					77 (60-98)					
Tetramer		100.40	99 (75%)			Homotetramer				
Graph	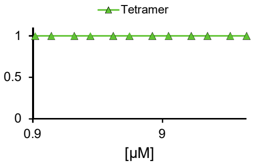		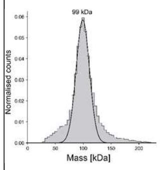		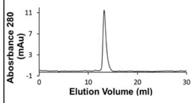			4ZNZ 1T75	highly expressed protein	

Upp- P0A8F0

Method	MS conc.	MS, kDa	MP, kDa (%)	SAXS, kDa	SEC, kDa	Swiss model calc.	Uniprot (kDa) and oligomerization	PDB	Protein abundance (copy number	
concentrations	1.875 - 40 µM		50nM	7-60 µM<br>0.1-0.6mg\ml	13.32 µM				Ref.1	Ref.2
Monomer							22.53		2780	4260
Dimer		44.95	44 (61%)		52 (40-66)					
Trimer										
Tetramer		89.89	89 (13%)	*I(0)* = 101.4 (σ=2.9)	88 (69-113)	Homotetramer				
Hexamer			131 (9%)		130 (101-166)					
Octamer			175 (5%)							
Graph	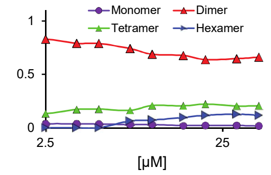		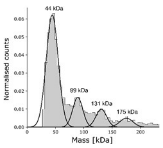		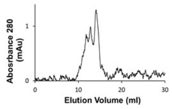		Homodimer or homotrimer in the absence of substrates, and homopentamer or homohexamer in the presence of substrates.	2EHJ	highly expressed protein	

SpeB- P60651

Method	MS conc.	MS, kDa	MP, kDa (%)	SAXS, kDa	SEC, kDa	Swiss model calc.	Uniprot (kDa) and oligomerization	PDB	Protein abundance (copy number	
concentrations	7.5 - 40 µM		110 nM	8-63 µM<br>0.3-2 mg\ml	9 µM				Ref.1	Ref.2
Monomer							33.56		3530	1063
Dimer			42 (25%)							
Trimer		100.54	105 (33%)		110 (86-140)					
Tetramer										
Hexamer		201.08	206 (15%)	*I(0)* = 175.5 (σ=7.3)	203 (159-260)	Hexamer				
Graph	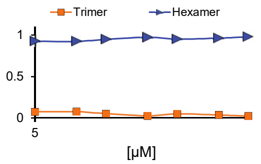		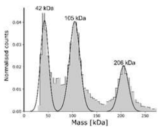		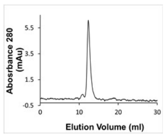			7LBA	highly expressed protein	

IspD- Q46893

Method	MS conc.	MS, kDa	MP, kDa (%)	SAXS, kDa	SEC, kDa	Swiss model calc.	Uniprot (kDa) and oligomerization	PDB	Protein abundance (copy number	
concentrations	7.5 - 40 µM			9-72 µM<br>0.23-1.9 mg\ml	12 µM				Ref.1	Ref.2
Monomer					43 (34-56)		25.61			
Dimer		51.35				Homodimer	Homodimer			
Trimer				*I(0)* = 99.6 (σ=3.5)	84 (66-108)					
Tetramer		102.71			133 (104-170)					
Hexamer										
Graph	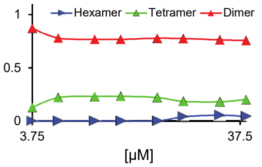		NA<br>Under the detection limit of MP		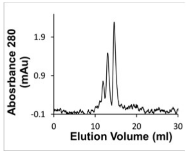			1VGT, 3N9W, 1i52	low expressed proteins	

BaeR- P69228

Method	MS conc.	MS, kDa	MP, kDa (%)	SAXS, kDa	SEC, kDa	Swiss model calc.	Uniprot (kDa) and oligomerization	PDB	Protein abundance (copy number	
concentrations	0.625 - 40 µM			13-107 µM<br>0.4-2.95 mg\ml	11 µM				Ref.1	Ref.2
Monomer		27.60		*I(0)* = 22.9 (σ=0.9)	25 (20-32)		27.66			
Dimer						dimer	dimer			
Graph	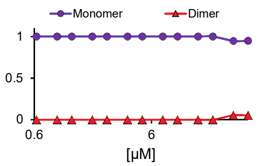		NA<br>Under the detection limit of MP		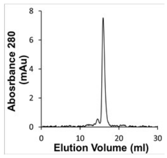			4B09	low expressed proteins	

AcuI P26646

Method	MS conc.	MS, kDa	MP, kDa (%)	SAXS, kDa	SEC, kDa	Swiss model calc.	Uniprot (kDa) and oligomerization	PDB	Protein abundance (copy number	
concentrations	0.625 - 40 μM		45 nM	4-29uM<br>0.125-1 mg\ml	134 nM			1O89<br>108C	Ref.1	Ref.2
Monomer		34.68	39 (35%)* fitted: 38(49%)	$I(0)$ =209.8 (σ=11.4)	56 (44-72)		34.73		186	882
Dimer		69.36	69 (47%) fitted: 67 (46%)			Homodimer	Homodimer		low expressed proteins	
Trimer										
Tetramer		104.04	134 (4%) fitted: 108(5%)		126 (98-161)					
Hexamer					201 (157-258)					
Graph	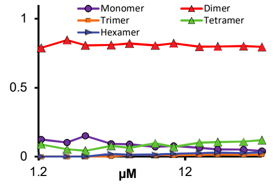		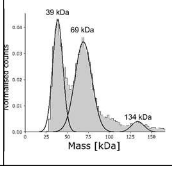		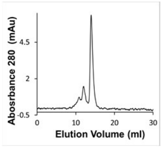					

PyrF P08244

Method	MS conc.	MS, kDa	MP, kDa (%)***	SAXS, kDa	SEC, kDa	Swiss model calc.	Uniprot (kDa) and oligomerization	PDB	Protein abundance (copy number	
concentrations	0.234- 40 μM		60 nM	10-81μM<br>0.3-2 mg\ml	11 μM			1EIX, 1L2U	Ref.1	Ref.2
Monomer		26.31		$I(0)$ = 57 (σ=4.1)	44 (35-57)		26.35		212	309
Dimer		52.61	58 (66%)			Homodimer	Homodimer		low expressed proteins	
Trimer										
Graph	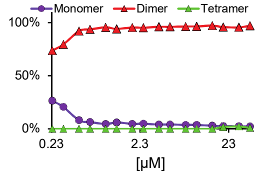		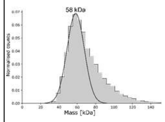		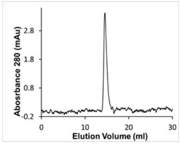					

ThiD P76422

Method	MS conc.	MS, kDa	MP, kDa (%)	SAXS, kDa	SEC, kDa	Swiss model calc.	Uniprot (kDa) and oligomerization	PDB	Protein abundance (copy number	
concentrations	5 - 40 μM		50 nM		10.5 μM			NA	Ref.1	Ref.2
Monomer		28.61			41 (32-52)		28.64		186	
Dimer		57.21	58 (81%)			Homodimer	Monomer		low expressed proteins	
Graph	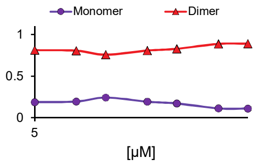		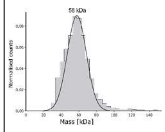		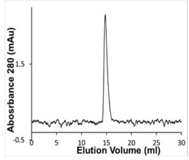					

NadE- P18843

Method	MS conc.	MS, kDa	MP, kDa (%)	SAXS, kDa	SEC, kDa	Swiss model calc.	Uniprot (kDa) and oligomerization	PDB	Protein abundance (copy number	
concentrations	0.938 - 40 μM		66 nM	9-74 μM<br>0.2-1.7 mg\ml	11 μM			1WXF, 1WXI	Ref.1	Ref.2
Monomer				$I(0)$ = 90.5 (σ=5.8)			27.16		746	598
Dimer		61.21	66 (83%)		46 (36-59)	Homodimer	Homodimer		low expressed proteins	
Tetramer										
Graph	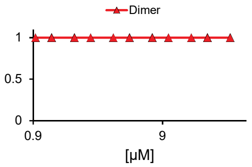		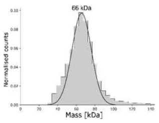		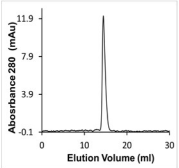					

Crp P0ACJ8

Method	MS conc.	MS, kDa	MP, kDa (%)	SAXS, kDa	SEC, kDa	Swiss model calc.	Uniprot (kDa) and oligomerization	PDB	Protein abundance (copy number	
concentrations	0.938 - 40 µM		48 nM		13 µM			2GZW, 3N4M, 5CIZ,1LB2	Ref.1	Ref.2
Monomer					31 (24-40)	Monomer, Homodimer	23.64		1980	3463
Dimer		47.16	48 (94%)				Dimer			
Graph	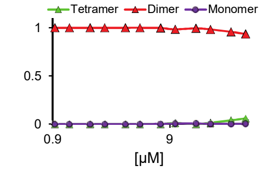		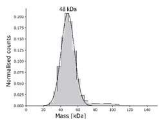	NA	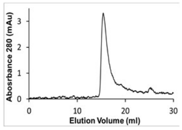				low expressed proteins	

Ref.1 Ishihama, Y., Schmidt, T., Rappsilber, J., Mann, M., Hartl, F. U., Kerner, M. J., & Frishman, D. (2008). Protein abundance profiling of the Escherichia coli cytosol. BMC Genomics, 9(1), 102.

Ref.2 Fauvet, Bruno, et al. "Bacterial Hsp90 mediates the degradation of aggregation-prone Hsp70-Hsp40 substrates preferentially by HslUV proteolysis." bioRxiv (2018): 451989.

emPAI-derived copy no/cell-

*Calculated using 1fL as the volume of the cell, protein concentration*avogadro no. * cell volume

Supplementary Table S3- Alpha Fold results of all 17 E.coli proteins					
**Oligomeric state**	**Sample name**	**pTM**	**plDDT**	**ipTM**	**PAE**
Monomer	2_NfuA	0.54	84.5		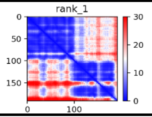
Dimer	2_NfuA_dimer	0.4		0.18	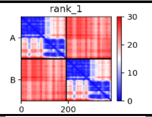
Trimer	2_NfuA_trimer	0.34		0.16	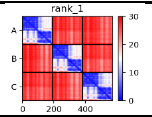
Tetramer	2_NfuA_tetramer	0.33		0.21	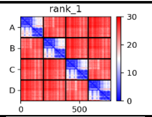
Hexamer	2_NfuA_hexamer	0.27		0.2	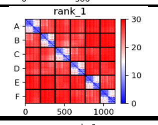
Monomer	3_NadK	0.83	91.4		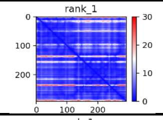
Dimer	3_NadK_dimer	0.92		0.91	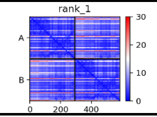
Trimer	3_NadK_trimer	0.8		0.75	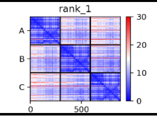
Tetramer	3_NadK_tetramer	0.89		0.88	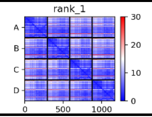
Hexamer	3_NadK_hexamer	0.39		0.33	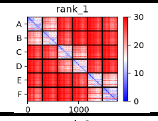
Monomer	4_FabG	0.87	97		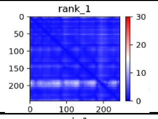
Dimer	4_FabG_dimer	0.95		0.94	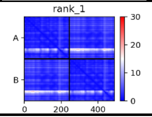
Trimer	4_FabG_trimer	0.81		0.77	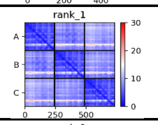
Tetramer	4_FabG_tetramer	0.94		0.93	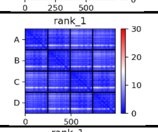
Hexamer	4_FabG_hexamer	0.78		0.76	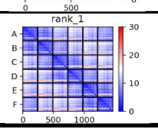

Monomer	5_DeoC	0.85	96.1		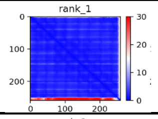
Dimer	5_DeoC_dimer	0.93		0.92	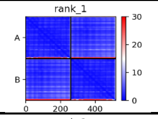
Trimer	5_DeoC_trimer	0.6		0.45	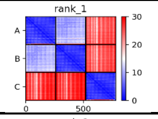
Tetramer	5_DeoC_tetramer	0.53		0.41	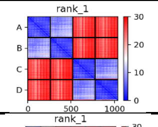
Hexamer	5_DeoC_hexamer	0.37		0.27	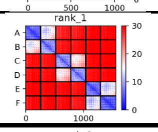
Monomer	6_eftS	0.8	94.2		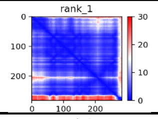
Dimer	6_eftS_dimer	0.61		0.42	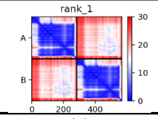
Trimer	6_eftS_trimer	0.41		0.2	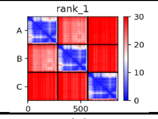
Tetramer	6_eftS_tetramer	0.42		0.28	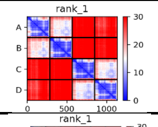
Hexamer	6_eftS_hexamer	0.34		0.25	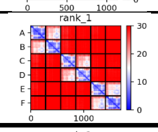
Monomer	7_SodA	0.86	97.8		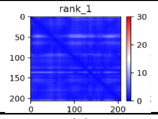
Dimer	7_SodA_dimer	0.95		0.93	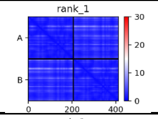
Trimer	7_SodA_trimer	0.66		0.51	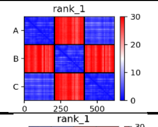
Tetramer	7_SodA_tetramer	0.52		0.37	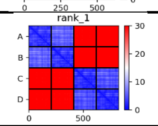
Hexamer	7_SodA_hexamer	0.39		0.29	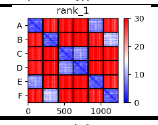
Monomer	9_gpmA	0.85	95.7		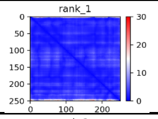
Dimer	9_gpmA_dimer	0.95		0.94	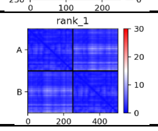

Trimer	9_gpmA_trimer	0.54		0.35	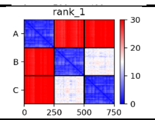
Tetramer	9_gpmA_tetramer	0.52		0.37	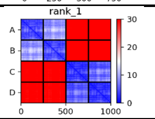
Hexamer	9_gpmA_hexamer	0.39		0.3	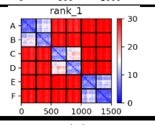
Monomer	11__can	0.84	95.3		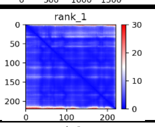
Dimer	11__can_dimer	0.94		0.94	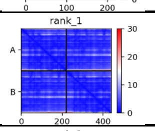
Trimer	11__can_trimer	0.9		0.87	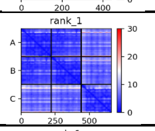
Tetramer	11__can_tetramer	0.95		0.94	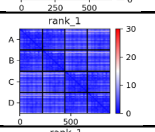
Hexamer	11__can_hexamer	0.44		0.36	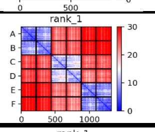
Monomer	12__upp	0.85	95.8		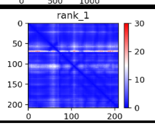
Dimer	12__upp_dimer	0.94		0.93	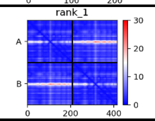
Trimer	12__upp_trimer	0.72		0.67	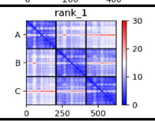
Tetramer	12__upp_tetramer	0.92		0.91	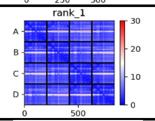
Hexamer	12__upp_hexamer	0.58		0.53	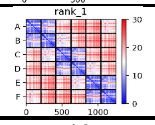
Monomer	13_speB	0.87	95.5		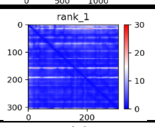
Dimer	13_speB_dimer	0.73		0.53	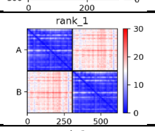
Trimer	13_speB_trimer	0.86		0.82	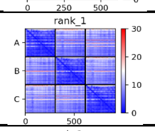
Tetramer	13_speB_tetramer	0.66		0.58	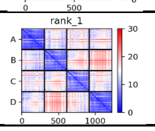

Hexamer	13_speB_hexamer	0.87		0.86	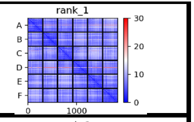
Monomer	14_ispD	0.83	92.5		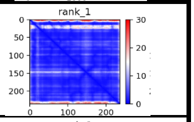
Dimer	14_ispD_dimer	0.89		0.9	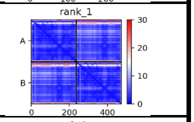
Trimer	14_ispD_trimer	0.56		0.43	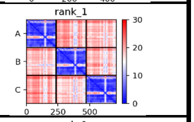
Tetramer	14_ispD_tetramer	0.48		0.38	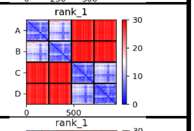
Hexamer	14_ispD_hexamer	0.36		0.29	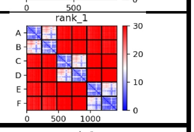
Monomer	15_BaeR	0.54	79.1		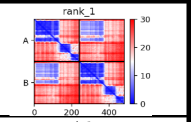
Dimer	15_BaeR_dimer	0.54		0.48	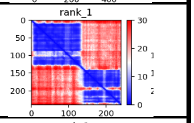
Trimer	15_BaeR_trimer	0.39		0.3	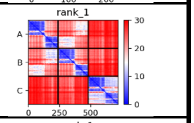
Tetramer	15_BaeR_tetramer	0.34		0.24	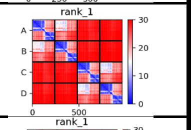
Hexamer	15_BaeR_hexamer	0.28		0.21	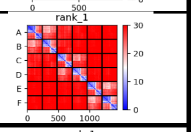
Monomer	16_AcuI	0.87	96.2		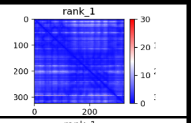
Dimer	16_AcuI_dimer	0.94		0.94	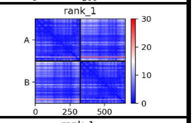
Trimer	16_AcuI_trimer	0.47		0.31	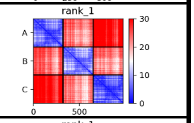
Tetramer	16_AcuI_tetramer	0.5		0.37	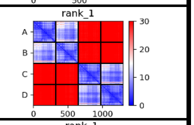
Hexamer	16_AcuI_hexamer	0.38		0.3	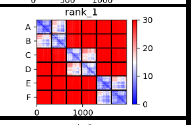
Monomer	17_pyrF	0.84	93.8		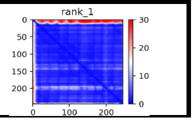

Dimer	17_pyrF_dimer	0.92		0.91	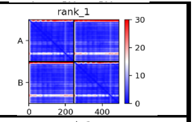
Trimer	17_pyrF_trimer	0.5		0.34	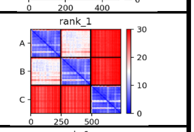
Tetramer	17_pyrF_tetramer	0.51		0.37	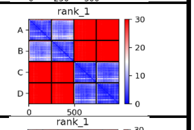
Hexamer	17_pyrF_hexamer	0.38		0.29	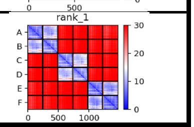
Monomer	19_thiD	0.86	93.7		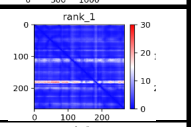
Dimer	19_thiD_dimer	0.95		0.94	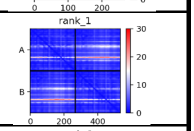
Trimer	19_thiD_trimer	0.52		0.36	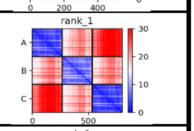
Tetramer	19_thiD_tetramer	0.52		0.41	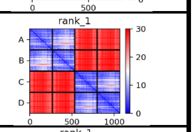
Hexamer	19_thiD_hexamer	0.4		0.31	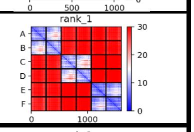
Monomer	23_nadE	0.86	95.4		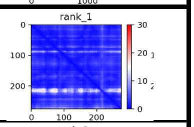
Dimer	23_nadE_dimer	0.95		0.95	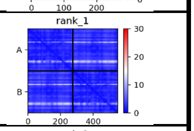
Trimer	23_nadE_trimer	0.54		0.41	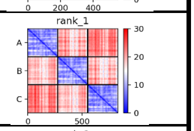
Tetramer	23_nadE_tetramer	0.52		0.39	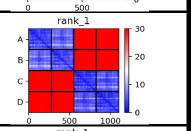
Hexamer	23_nadE_hexamer	0.4		0.3	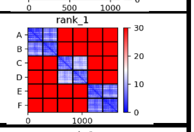
Monomer	27_crp	0.8	94.4		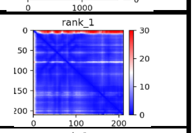
Dimer	27_crp_dimer	0.92		0.92	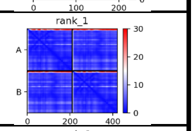
Trimer	27_crp_trimer	0.83		0.81	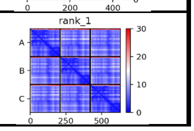

Tetramer	27_crp_tetramer	0.5		0.37	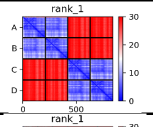
Hexamer	27_crp_hexamer	0.38		0.28	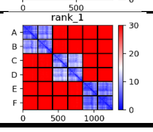
